# Tumor suppressor BTG1 promotes PRMT1-mediated ATF4 function in response to cellular stress

**DOI:** 10.18632/oncotarget.6519

**Published:** 2015-12-09

**Authors:** Laurensia Yuniati, Laurens T. van der Meer, Esther Tijchon, Dorette van Ingen Schenau, Liesbeth van Emst, Marloes Levers, Sander A.L. Palit, Caroline Rodenbach, Geert Poelmans, Peter M. Hoogerbrugge, Jixiu Shan, Michael S. Kilberg, Blanca Scheijen, Frank N. van Leeuwen

**Affiliations:** ^1^ Laboratory of Pediatric Oncology, Radboud Institute for Molecular Life Science, Radboud University Medical Center, Nijmegen, The Netherlands; ^2^ Department of Cognitive Neuroscience, Donders Institute for Brain, Cognition and Behaviour, Radboud University Medical Center, Nijmegen, The Netherlands; ^3^ Department of Human Genetics, Radboud University Medical Center, Nijmegen, The Netherlands; ^4^ Department of Molecular Animal Physiology, Donders Institute for Brain, Cognition and Behaviour, Radboud University, Nijmegen, The Netherlands; ^5^ Prinses Maxima Center for Pediatric Oncology, De Bilt, The Netherlands; ^6^ Department of Biochemistry and Molecular Biology, University of Florida College of Medicine, Gainesville, FL, USA

**Keywords:** BTG1, leukemia, ATF4, cellular stress, PRMT1

## Abstract

Cancer cells are frequently exposed to physiological stress conditions such as hypoxia and nutrient limitation. Escape from stress-induced apoptosis is one of the mechanisms used by malignant cells to survive unfavorable conditions. *B-cell Translocation Gene 1* (*BTG1*) is a tumor suppressor that is frequently deleted in acute lymphoblastic leukemia and recurrently mutated in diffuse large B cell lymphoma. Moreover, low BTG1 expression levels have been linked to poor outcome in several solid tumors. How loss of BTG1 function contributes to tumor progression is not well understood. Here, using *Btg1* knockout mice, we demonstrate that loss of *Btg1* provides a survival advantage to primary mouse embryonic fibroblasts (MEFs) under stress conditions. This pro-survival effect involves regulation of Activating Transcription Factor 4 (ATF4), a key mediator of cellular stress responses. We show that BTG1 interacts with ATF4 and positively modulates its activity by recruiting the protein arginine methyl transferase PRMT1 to methylate ATF4 on arginine residue 239. We further extend these findings to B-cell progenitors, by showing that loss of Btg1 expression enhances stress adaptation of mouse bone marrow-derived B cell progenitors. In conclusion, we have identified the BTG1/PRMT1 complex as a new modifier of ATF4 mediated stress responses.

## INTRODUCTION

During cancer progression, tumor cells are exposed to physiological stresses, such as hypoxia and nutrient limitation, either as a result of aberrant proliferation or chemotherapy intervention [1]. Depending on the cell type as well as the intensity or duration of stress, tumor cells will either employ survival mechanisms or initiate programmed cell death. Activating Transcription Factor 4 (ATF4) is a master regulator of the stress response pathway that is activated upon micro-environmental stresses including ER stress, amino acid limitation, fluctuations in nutrient availability, hypoxia and oxidative stress [2–8]. In general, stressed cells turn on the amino acid response (AAR) (triggered by amino acid limitation) or the unfolded protein response (UPR) (triggered by endoplasmic reticulum (ER) stress) by activating GCN2 or PERK protein kinases, respectively. The phosphorylation events mediated by these kinases repress global protein synthesis but paradoxically increase the translation of a subset of mRNAs, including that of ATF4 [2, 3, 9]. This member of the basic-region leucine zipper (bZIP) transcription factor family both directly and indirectly promotes the expression of hundreds of genes involved in metabolism, protein synthesis and nutrient homeostasis. ATF4 targets also include genes that promote apoptosis (e.g., *DDIT3, TRB3*) [2, 10], which allows ATF4 to establish a tight balance between salvage and cell death, depending on the ability of the cells to adapt to environmental challenges. Consequently, ATF4-mediated stress responses govern metabolic and oxidative adaptation in various cell types such as neurons, osteoblasts, hepatocytes and hematopoietic stem cells [6, 11–13]. Moreover, this pathway is deregulated in a variety of cancers, including fibrosarcoma, neuroblastoma, multiple myeloma and breast cancer, which allows cancer cells to sustain growth under unfavorable circumstances [14–17].

The B-cell Translocation Gene 1 (*BTG1*) belongs to the BTG/Tob anti-proliferation gene family [18, 19]. The protein product expressed by the *BTG1* gene is broadly expressed and regulates various biological and cellular processes including cell cycle regulation, control of proliferation and differentiation, induction of apoptosis, transcriptional activation, and regulation of mRNA stability [19–22]. BTG1 has no intrinsic catalytic activity but rather acts as an adaptor protein for other regulatory molecules. For instance, BTG1 was shown to act as a transcriptional co-regulator by interacting with and recruiting protein arginine methyltransferase 1 (PRMT1) to transcription factor complexes [23]. We and others have shown that *BTG1* is affected by monoallelic deletion in about 9% of childhood pre-B acute lymphoblastic leukemia (B-ALL) cases [24, 25], and that existing or novel *BTG1* copy number aberrations can be detected at relapse [26, 27]. In addition, *BTG1* is among recurrently mutated genes detected in B-cell lymphoma samples [28, 29]. Furthermore, low *BTG1* mRNA expression is associated with poor outcome or tumor metastasis in several solid tumors [30–35]. How tumor cells benefit from loss of BTG1 function remains to be elucidated.

Given the fact that the expression of *BTG1*, and its closest family member *BTG2*, are increased by a wide range of stress-inducing stimuli, such as chemotherapeutic compounds and genotoxic agents [36, 37], we hypothesized that both proteins may act to modulate cellular stress response pathways. In this report, we investigated the involvement of BTG1 and BTG2 in regulating ATF4, a critical regulator of the integrated stress response both in normal and cancer cells [8, 9, 14, 38, 39]. We demonstrate that loss of BTG1, but not BTG2, protects cells from cellular stress-mediated apoptosis by reducing PRMT1-mediated arginine methylation of ATF4.

## RESULTS

### Loss of BTG1 promotes survival in response to cellular stress

**Figure 1:**
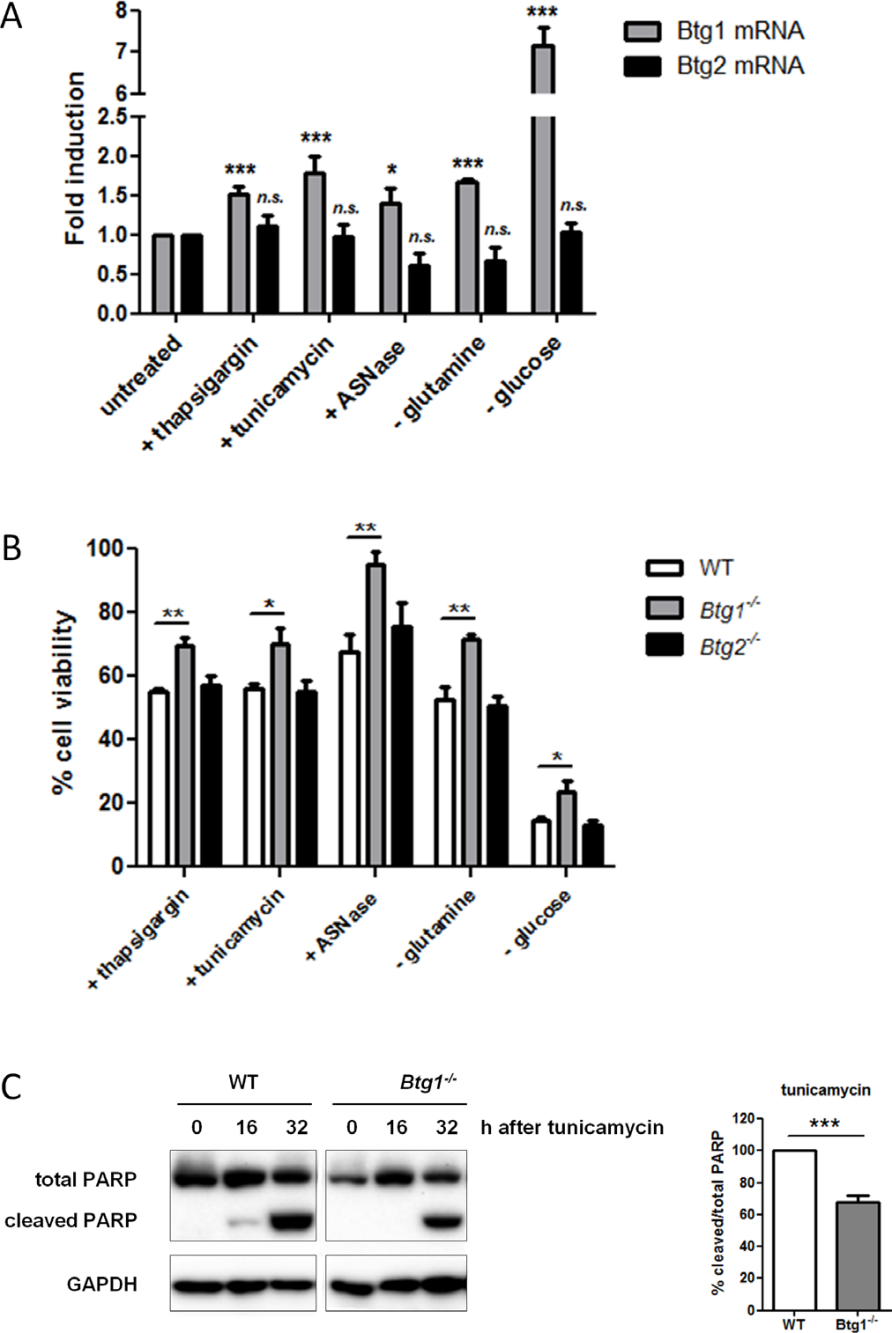
BTG1 affects cellular responses to stress (**A**) *Btg1* mRNA is upregulated in response to various stress stimuli. WT MEFs were treated with various stressors and mRNA levels of *Btg1* and *Btg2* relative to untreated cells are shown. Bars represent average data from four independent experiments ± SEM. (**B**) BTG1, but not BTG2 expression, is required for survival under cellular stress conditions. WT, *Btg1*^−/−^ and *Btg2*^−/−^ MEFs were challenged with ER stress-inducing drugs (thapsigargin, tunicamycin), glucose depletion, and amino acid limitation (glutamine starvation, Asparaginase (ASNase)). Metabolic activity was determined by MTS assay and cell survival relative to untreated cells (set at 100%) is shown. Bars represent average data from four independent experiments ± SEM. (**C**) Loss of BTG1 protects cells from stress-induced apoptosis. WT and *Btg1*^−/−^ MEFs were stressed with tunicamycin and apoptosis was measured by western blot for cleaved PARP protein. Bars represent average data from three independent experiments ± SEM. *P*-values are indicated with ***P < 0.001, **P < 0.01 and *P < 0.05 (two-tailed *t*-test).

*BTG/TOB* family members have been implicated in the inhibition of cell growth and the induction of apoptosis in a variety of model systems [19, 40–45]. Here, we applied a range of compounds to induce ATF4-mediated cellular stress responses (drug-induced ER stress, amino acid limitation and nutrient depletion) and found the *Btg1* mRNA to be upregulated in mouse embryonic fibroblasts (MEFs) in response to these stressors (Figure 1A). In contrast, the *Btg2* transcript remained unaffected under these conditions. To establish a role for BTG1 in the ATF4 stress response pathway, we challenged primary MEFs deficient for *Btg1* or *Btg2* with amino acid and nutrient limitation as well as ER stress-inducing compounds. We quantified cell viability before and after stress treatment using an MTT-based viability assay, and the percentage of cell survival was calculated relative to untreated cells (set to 100%). There was no significant difference in the growth properties between cells derived from each genotype under normal conditions (data not shown). We observed that while *Btg2* knockout cells cope with stress similarly to wild-type (WT) cells, loss of *Btg1* provides a significant survival benefit (Figure 1B). By measuring the amount of lactate dehydrogenase (LDH) released into medium, as a measure of cell death, we confirmed that *Btg1* knockout cells are more resistant to cellular stress in comparison to WT cells (Supplementary Figure S1). In tunicamycin treated cells, this increase in survival reflects reduced induction of apoptosis, as indicated by the amount of cleaved PARP protein (Figure 1C). From these experiments, we conclude that, at least in this particular cell type, loss of BTG1, but not BTG2, enhances cellular survival to ATF4-inducing cellular stressors.

### BTG1 positively regulates the activity of transcription factor ATF4

**Figure 2:**
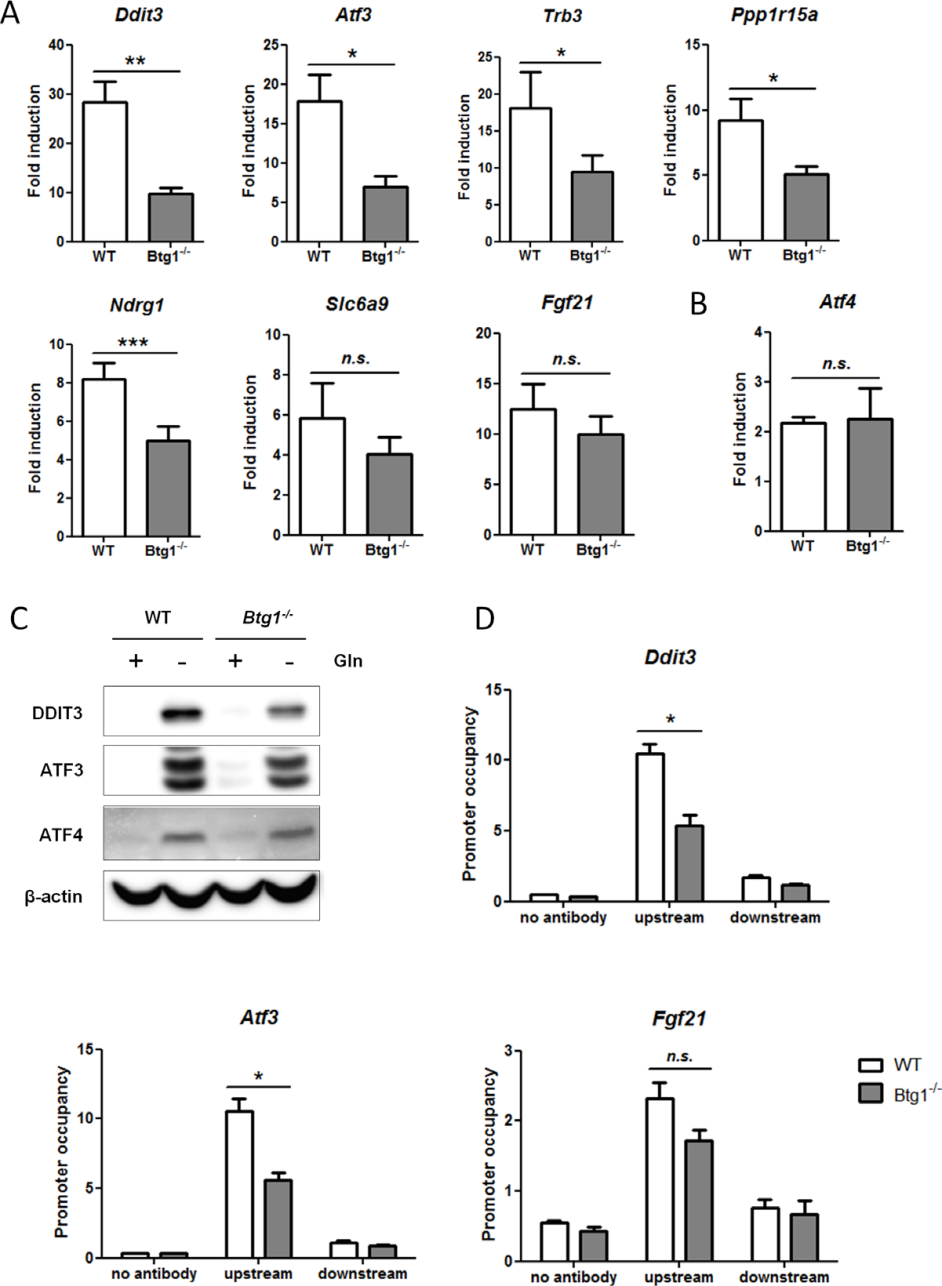
Loss of BTG1 negatively affects ATF4-mediated gene expression (**A**) Reduced expression of ATF4 target genes in *Btg1*^−/−^ cells upon glutamine depletion. WT and *Btg1*^−/−^ MEFs were stressed with glutamine starvation for 16 hours and qPCR was performed to measure the expression level of the seven ATF4 targets identified by gene expression analysis (Table 3). Data is presented as fold induction of mRNA (expression level of untreated samples were set to 1). Bars represent average data from four independent experiments ± SEM. *P*-values are indicated with ***P < 0.001, **P < 0.01 and *P < 0.05 (two-tailed paired *t*-test). (**B**) The expression level of Atf4 itself is not affected by the absence of Btg1. (**C**) The expression of ATF4 target genes in *Btg1*^−/−^ cells is also attenuated at protein level. Western blot shows the protein expression of two ATF4 targets following glutamine starvation. (**D**) ATF4 occupancy on target gene promoters is affected by loss of BTG1. WT and *Btg1*^−/−^ MEFs were stressed by glutamine starvation for 16 hours and ChIP was performed using an ATF4 antibody to determine ATF4 binding on the promoters of *Ddit3*, *Atf3* and *Fgf21*. IP without antibody served as negative control. For each target gene, qPCR was performed using primers which recognize the ATF4 binding site (upstream) and a control region around 1.5 kb further (downstream). Bars represent average data from three independent experiments ± SEM. *P*-values are indicated with *P < 0.05 (two-tailed paired *t*-test).

To examine how the presence or absence of BTG1 affects cellular stress responses, we performed gene expression analyses comparing WT and *Btg1*^−/−^ MEFs subjected to glutamine starvation. We chose glutamine deprivation for these analyses since glutamine limitation is a physiological stressor known to activate ATF4 and frequently encountered by aberrantly proliferating cancer cells [46–48]. Using the Ingenuity software package (IPA) (see Materials and Methods), we identified transcriptional regulators that were either activated or inhibited during this nutrient limitation in both WT and *Btg1*^−/−^ cells (Table 1). This analysis confirmed that ATF4 is among the top transcription regulators activated by glutamine deprivation. We subsequently examined whether ATF4-mediated gene expression was different between the two genotypes, by comparing the fold induction of ATF4 target genes following glutamine starvation in WT versus *Btg1* knockout cells. Using predefined criteria (see Materials and Methods; fold change (FC) ≥ |3|), we identified twelve regulated genes (Table 2). By including only genes with established prediction state (activated or inhibited), we found that seven out of twelve ATF4 target genes, regulated by glutamine limitation, were selectively lower expressed in *Btg1*^−/−^ MEFs (Table 3). We replicated and validated these findings by real-time quantitative PCR (qPCR) and found that for at least five ATF4 target genes (*Ddit3, Atf3, Trb3, Ppp1r15a, Ndrg1*), upregulation by glutamine deprivation was significantly suppressed in *Btg1* knockout cells in comparison to WT cells (Figure 2A). Moreover, the attenuated response of these genes was specific to the stress treatment, since the expression of these genes under non-starved conditions was comparable between WT and *Btg1*^−/−^ cells, except for *Ddit3*, which showed a modest increase in basal expression in the knockout setting (data not shown). Importantly, *Atf4* mRNA induction following glutamine starvation was not affected by loss of BTG1 protein expression, suggesting that ATF4 activity rather than expression is subject to regulation by BTG1 (Figure 2B). For two of these targets (*Ddit3* and *Atf3*), we confirmed at the protein level that loss of BTG1 protein expression suppresses the extent of induction in response to glutamine starvation (Figure 2C). To demonstrate that loss of BTG1 reduces the ability of ATF4 to activate these target genes, chromatin immunoprecipitation (ChIP) assays were performed, comparing lysates from primary WT and *Btg1*^−/−^ MEFs subjected to glutamine starvation. Consistent with a role for BTG1 in the regulation of ATF4 activity, we observed a decrease in ATF4 binding to the promoter regions of *Ddit3* and *Atf3*, and to a lesser extent to the promoter of *Fgf21*, in the *Btg1*^−/−^ MEFs relative to WT controls (Figure 2D). Together, these experiments demonstrate that BTG1 affects cellular responses to stress, by positively regulating a subset of ATF4 target genes at the transcriptional level.

**Table 1:** Ingenuity upstream regulator analysis of the transcription factors regulating the expression of the genes that we found to be differentially expressed after glutamine starvation in WT and *Btg1*^−/−^ MEFs

WT MEFs			
Transcription regulator	Regulated genes	Predicted activation state (Z-score)*	*P*-value**
ATF4	*Areg, Atf3, Ccl2, Ddit3, Fgf21, Gch1, Lgals3, Mt2a, Ndrg1, Nid2, Pmp22, Ppp1r15a, Ptx3, Sars, Slc1a5, Slc6a9, Slc7a3, Stc2, Tnfsf11, Trib3*	2,509 (activated)	1.00E–14
NUPR1	*Adm, Areg, Aspm, Atf3, Atp8b1, Bub1b, C9orf91, Cdca3, Cenpi, Ckap2l, Col3a1, Cxadr, Ddit3, Dhcr24, Eno2, Fam83g, Gadd45a, Gch1, Gpr1, Gsta4, Hdac8, Hist1h2ag, Hist1h2ah, Il13ra1, Kif11, Kif23, Kif2c, Lmnb1, Myh10, Ndrg1, Nupr1, Plk1, Pmp22, Ppp1r15a, Spc24, Tmem136, Trib3*	4,014 (activated)	4.62E–14
KDM5B	*Bub1b, Ccne1, Cdca3, Ddit3, Dhcr24, Dlgap5, Fabp5, Gadd45a, Gal, Gjb2, Insig1, Kif2c, Mcam, Ncaph, Nedd9, Pbk, Smox, Top2a*	4,111 (activated)	1.06E–12
MYC	*Acta1, Adm, Anxa6, Aqp1, Birc5, Bub1b, Ccne1, Ccne2, Cd274, Cdc20, Chka, Col15a1, Col2a1, Col3a1, Cryab, Ddb2, Ddit3, Fabp5, Fads2, Fam129a, Fgf5, Fos, Foxm1, Gadd45a, Gamt, H19, Hmox1, Id2, Idh2, Iqgap2, Lum, Lxn, Ndrg1, Peg3, Plau, Plk1, Pmp22, Ppp1r15a, Rrm2, Sfrp1, Slc1a5, St3gal1, Thbs1, Tnfsf11, Vldlr*	−2,607 (inhibited)	2.53E–11
SMAD7	*Areg, Ccl2, Ccne1, Col2a1, Col3a1, Fst, Gadd45a, Hmox1, Itgbl1, Ltbp2, Nid2, Sema3f, Tagln, Tgfb2, Tgfb3, Vdr*	2,353 (activated)	3.36E–10

*Btg1*^−/−^ MEFs			
Transcription regulator	Regulated genes	Predicted activation state (Z-score)*	*P*-value**
NUPR1	*Areg, As3mt, Atf3, Aurka, C9orf91, Cdca3, Ckap2l, Cxadr, Ddit3, Dhcr24, Espl1, Fam83g, Gadd45a, Gpr1, Hist1h2ag, Hist1h2ah, Kif11, Kif23, Kif2c, Lmnb1, Ndrg1, Plk1, Ppp1r15a, Spc24, Trib3, Uprt*	3,806 (activated)	3.20E–14
KDM5B	*Aurka, Cdca3, Ddit3, Dhcr24, Dlgap5, Gadd45a, Gjb2, Insig1, Kif2c, Mcam, Ncaph, Pbk, Top2a*	3,591 (activated)	9.53E–12
ATF4	*Areg, Atf3, Ddit3, Fgf21, Mt2a, Ndrg1, Ppp1r15a, Ptx3, Slc6a9, Stc2, Tnfrsf11a, Tnfsf11, Trib3*	2,376 (activated)	1.19E–11
TP53	*Areg, Atf3, Aurka, Birc5, Bok, Ccne2, Cdc20, Chst12, Cxcl1, Cyp51a1, Ddit3, Dhcr24, Dlgap5, Espl1, Foxm1, Gadd45a, Hmox1, Kcnj4, Kif23, Mcam, Melk, Mis18bp1, Ncaph, Ndrg1, Pbk, Plau, Plk1, Ppp1r15a, Rad51ap1, Rrm2, Stard4, Top2a, Trib3*	2,473 (activated)	3.02E–09
CTNNB1	*Aoc3, Birc5, Bmp4, Ccne2, Cyp51a1, Eno3, Gjb2, Grem1, Kif23, Lck, Mylk, Phlda2, Plau, Plk1, Prss35, Sfrp1, Sqstm1, Tgfb3, Tnfsf11, Wnt4*	−2,023 (inhibited)	2.38E–08

* Z-score is calculated based on how many times the expression direction of the differentially expressed genes matches the expression direction that has been proven to be caused by that transcriptional regulator according to the Ingenuity Knowledge Base. It is calculated with a formula reflecting the ‘significant pattern match’ of expression up/down regulation, and significance is generally attributed to Z-scores ≥ 2 (‘activated’ transcriptional regulator) or ≤ −2 (‘inhibited’ transcriptional regulator).

** This *P*-value indicates whether there is a statistically significant overlap between the differentially expressed genes and the genes of which the expression is known to be regulated by a given transcription factor according to the Ingenuity Knowledge Base, which is based on information from the published literature as well as many other sources, including gene expression and gene annotation databases (*http://www.ingenuity.com*). It is calculated with the Fisher's exact test, and significance is generally attributed to *P*-values < 0.01.

The top five transcription factors that have a predicted activation or inhibition state with a Z-score ≥ |2| are shown.

**Table 2:** ATF4 target genes regulated in response to glutamine starvation in WT and *Btg1*^−/−^ MEFs

Genes	Prediction	FC WT	FC*Btg1*^−/−^
*Areg*	Affected	10,777	6,514
*Atf3*	Activated	25,831	15,950
*Ddit3*	Activated	19,262	7,715
*Fgf21*	Activated	9,716	4,596
*Mt2*	Affected	9,836	5,096
*Ndrg1*	Activated	7,593	5,557
*Ppp1r15a*	Activated	7,201	4,689
*Ptx3*	Activated	4,493	4,489
*Slc6a9*	Activated	5,943	3,709
*Stc2*	Affected	5,465	6,912
*Tnfsf11*	Affected	−7,817	−3,804
*Trb3*	Activated	9,441	6,473

Abbreviations: FC = fold change. Prediction is based on whether the expression of the Atf4 target gene is upregulated or downregulated according to the literature (i.e., whether Atf4 is predicted to be activated or inhibited).

### ATF4 is a substrate for PRMT1-mediated methylation

**Figure 3:**
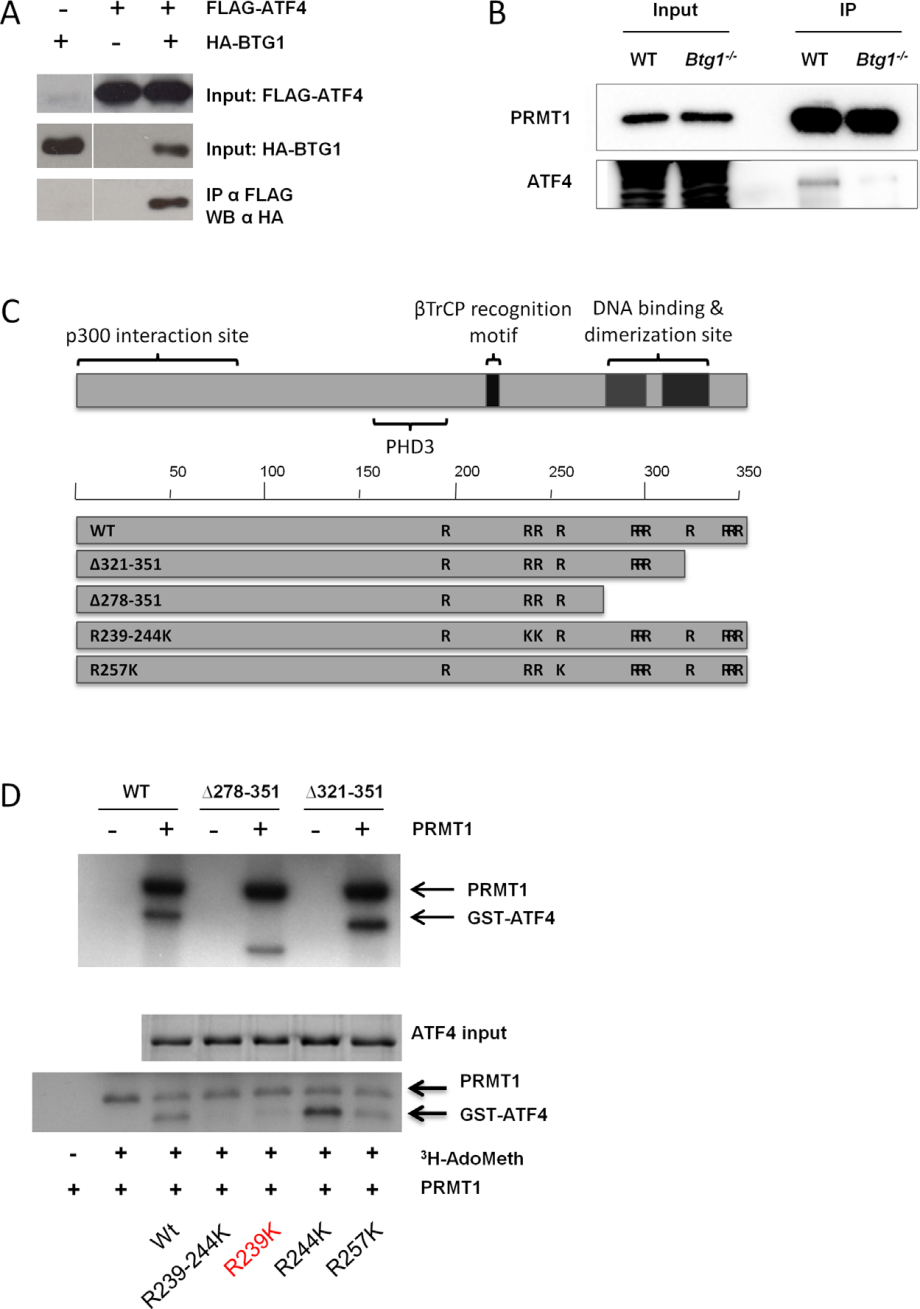
BTG1 facilitates PRMT1 binding to and methylation of ATF4 (**A**) BTG1 binds to ATF4. HEK293 cells were transfected with expression plasmids encoding HA-ATF4 and FLAG-BTG1 and treated for 24 hrs with 5 μM of the proteasome inhibitor MG132. Protein lysates were generated and subjected to immunoprecipitation (IP) with FLAG antibody (Ab). Immunoblot demonstrates expression of BTG1 using a FLAG-Ab. (**B**) PRMT1 binds to ATF4 in a BTG1-dependent manner. WT and *Btg1*^−/−^ MEFs were treated with glutamine starvation for 16 h. Protein lysates were generated and subjected to IP with PRMT1 Ab. Immunoblot demonstrates expression of ATF4. (**C**) Mapping of arginine residues found in ATF4. (**D**) ATF4 is methylated by PRMT1 at amino acid (aa) residue 239. GST-purified ATF4 WT and various ATF4 deletion mutants (top panel) and arginine mutants (bottom panel) were subjected to *in vitro* methylation assays together with purified PRMT1 and S-adenosyl methionine as a methyl donor. Proteins were resolved by SDS-PAGE, stained with Coomassie blue (ATF4 input), dried and analyzed by autofluorography. Mutation of arginine 239 abolishes methylation of ATF4.

To further explore how BTG1 controls ATF4 activity, we performed co-immunoprecipitation (co-IP) assays from HEK293 cells made to co-express recombinant (FLAG-tagged)-BTG1 and (HA-tagged)-ATF4, showing that BTG1 complexes with ATF4 (Figure 3A). Since BTG1 has no known enzymatic function, we hypothesized that there may be an additional co-factor in the BTG1-ATF4 complex that modulates ATF4 activity in a BTG1 dependent manner. p300 and pCAF are two known activators of ATF4 [49, 50]. By co-IP assays in HEK293, we determined whether the interaction between ATF4 and either p300 or pCAF was affected upon co-expression of BTG1, but no differences were observed (data not shown). Another candidate cofactor is PRMT1, a known binding partner for BTG1 [23, 51]. PRMT1 induces protein arginine methylation, a post-translational modification involved in the regulation of transcription factor activity [52, 53]. Co-IP assays from primary MEFs confirmed that endogenous PRMT1 and ATF4 are in a complex, which appears to be dependent on the presence of BTG1 (Figure 3B). To complement these findings, we re-expressed BTG1 in Btg1^−/−^ MEFs confirming that BTG1 expression is indispensable for the binding between ATF4 and PRMT1 (Supplementary Figure S2).

To further analyze if and how BTG1-dependent binding of PRMT1 to ATF4 modulates its function, we deleted one or more arginine residues present in ATF4 to locate potential arginine residue(s) targeted by PRMT1-mediated methylation (Figure 3C). We cloned ATF4 WT and ATF4 arginine mutants into expression plasmids and purified these proteins by their GST tags. By *in vitro* methylation assays combining purified ATF4, purified PRMT1 and ^35^S-adenosylmethionine as a methyl donor, we demonstrated that ATF4 is indeed methylated by PRMT1 (Figure 3D, top panel). While the deletion mutants did not show impaired methylation, we identified arginine residue 239 as the most prominent arginine methylated site in ATF4 (Figure 3D, bottom panel). We conclude from these experiments that BTG1 promotes PRMT1-mediated arginine methylation of ATF4.

### PRMT1-mediated methylation potentiates ATF4 transcriptional activity

**Figure 4:**
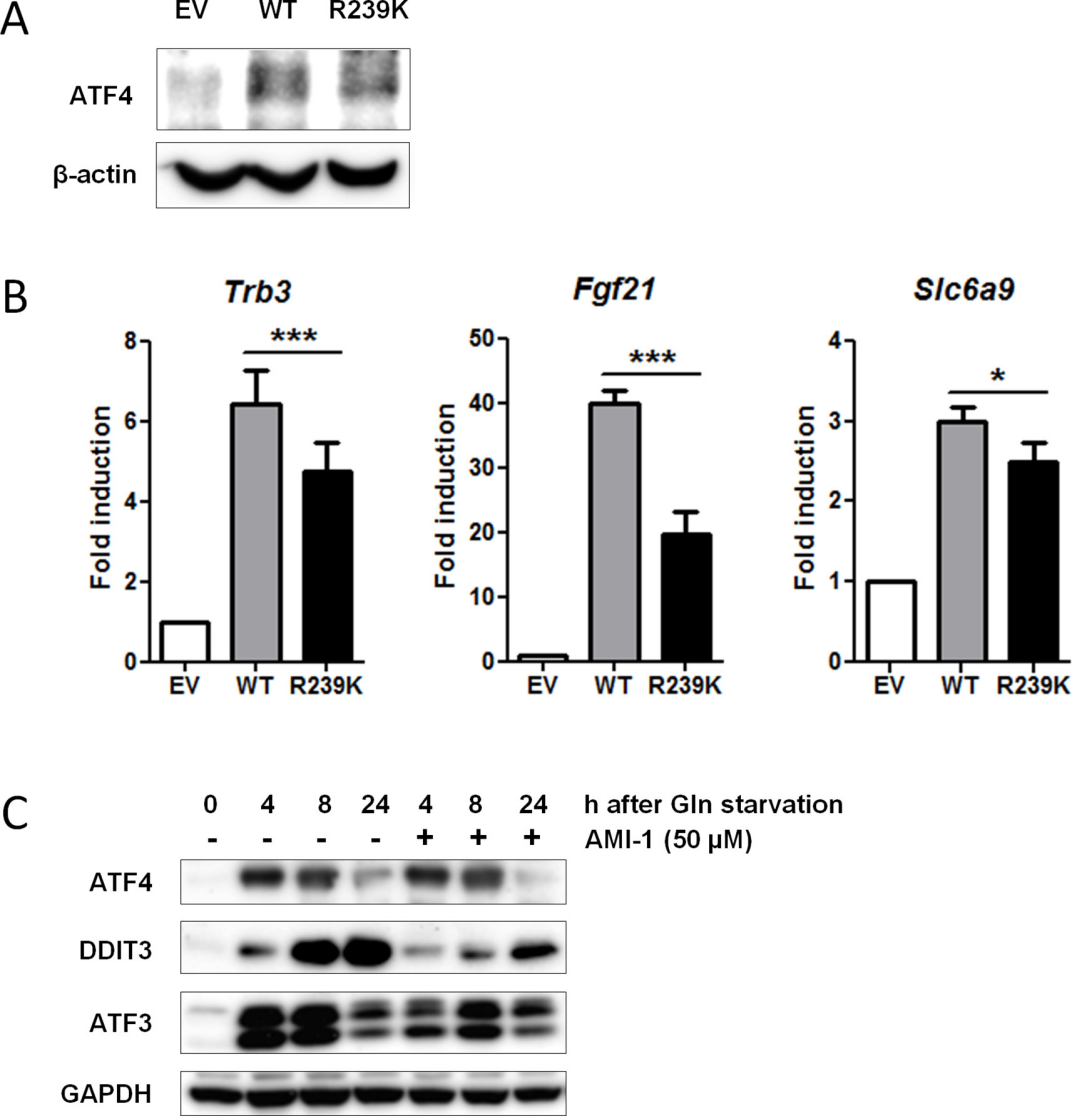
Loss of PRMT1-mediated methylation at residue R239 attenuates ATF4 function (**A**) Both WT and ATF4-R239K are equally expressed. Non-methylated ATF4-R239K was generated by overlap extension PCR (see Materials and Methods). WT and ATF4-R239K as well as an empty vector (EV) were retrovirally transduced into immortalized *Atf4*^−/−^ MEFs. Protein lysates were generated and subjected to IP with ATF4Ab. Immunoblot demonstrates expression of ATF4. β-actin levels were used as a loading control. (**B**) ATF4 methylation potentiates its activity. EV, *ATF4-WT* and *ATF4-R239K* complemented MEFs were subjected to qPCR to determine expression of ATF4 targets identified by gene expression analysis (Table 3). Data are presented as fold induction of mRNA (expression level of untreated EV MEFs were set to 1). Bars represent average data from four independent experiments ± SEM. *P*-values are indicated with ***P < 0.001, **P < 0.01 and *P < 0.05 (two-tailed paired *t*-test). (**C**) WT MEFs were treated with 50 μM AMI-1 in the presence and absence of glutamine and western blot was performed for several ATF4 targets. GAPDH levels were used as a loading control.

To establish if and how ATF4 methylation by PRMT1 affects its transcriptional activity, we complemented SV40 immortalized *Atf4*^−/−^ MEFs with human wild type *ATF4 (ATF4-WT*) or methylation mutant *ATF4-R239K*. As a negative control, an empty vector was introduced into these *Atf4* knockout MEFs. Both *ATF4-WT* and *ATF4-R239K* were expressed at comparable levels as shown by qPCR (Supplementary Figure S3A), although expression levels were approximately half of that of endogenous *ATF4* present in WT cells (data not shown). Immunoprecipitations revealed that the expression and stability of both proteins was comparable (Figure 4A). Subsequently, qPCR was performed in these *ATF4*-complemented MEFs to determine the transcript levels of seven ATF4 target genes listed in Table 3. Using this system, complementation of *ATF4-WT* and *ATF4-R239K* was sufficient to upregulate the expression of *Trb3, Fgf21* and *Slc6a9*, but the extent of upregulation was significantly reduced for all three target genes when the R293K mutant was used (Figure 4B). *ATF4* complementation did not affect target gene regulation of the four other *ATF4* target genes (*Ddit3, Atf3, Ndrg1, Ppp1r15a*), suggesting that these genes are not exclusively dependent on ATF4 function in this immortalized model cell line (Supplementary Figure S3B). To further assess the contribution of PRMT1-mediated methylation to ATF4 function, we inhibited PRMT1 activity with a specific molecular inhibitor, AMI-1 [54]. We applied this methyl transferase inhibitor to WT MEFs and determined by Western blotting that induction of at least two ATF4 targets, DDIT3 and ATF3, was repressed upon glutamine deprivation, while expression of ATF4 protein remained unaffected (Figure 4C). These findings indicate that arginine methylation of ATF4 by PRMT1 enhances its activity towards a selective set of target genes.

**Table 3:** ATF4 target genes differentially activated under glutamine starvation in WT versus *Btg1*^−/−^ MEFs

Genes	FC WT	FC*Btg1^−/−^*
*Atf3*	25,831	15,950
*Ddit3*	19,262	7,715
*Fgf21*	9,716	4,596
*Ndrg1*	7,593	5,557
*Ppp1r15a*	7,201	4,689
*Slc6a9*	5,943	3,709
*Trb3*	9,441	6,473

Abbreviations: FC = fold change; Atf3 = Activating transcription factor 3; Ddit3 = DNA damage-inducible transcript 3; Fgf21 = Fibroblast growth factor 21; Ndrg1 = N-Myc downstream regulated 1; Ppp1r15a = Protein phosphatase 1, regulatory subunit 15A; Slc6a9 = Solute carrier family 6 (neurotransmitter transporter, glycine), member 9; Trb3 = Tribbles pseudokinase 3.

### BTG1 and ATF4 are components of the cellular stress response in bone marrow-derived B cell progenitors

**Figure 5:**
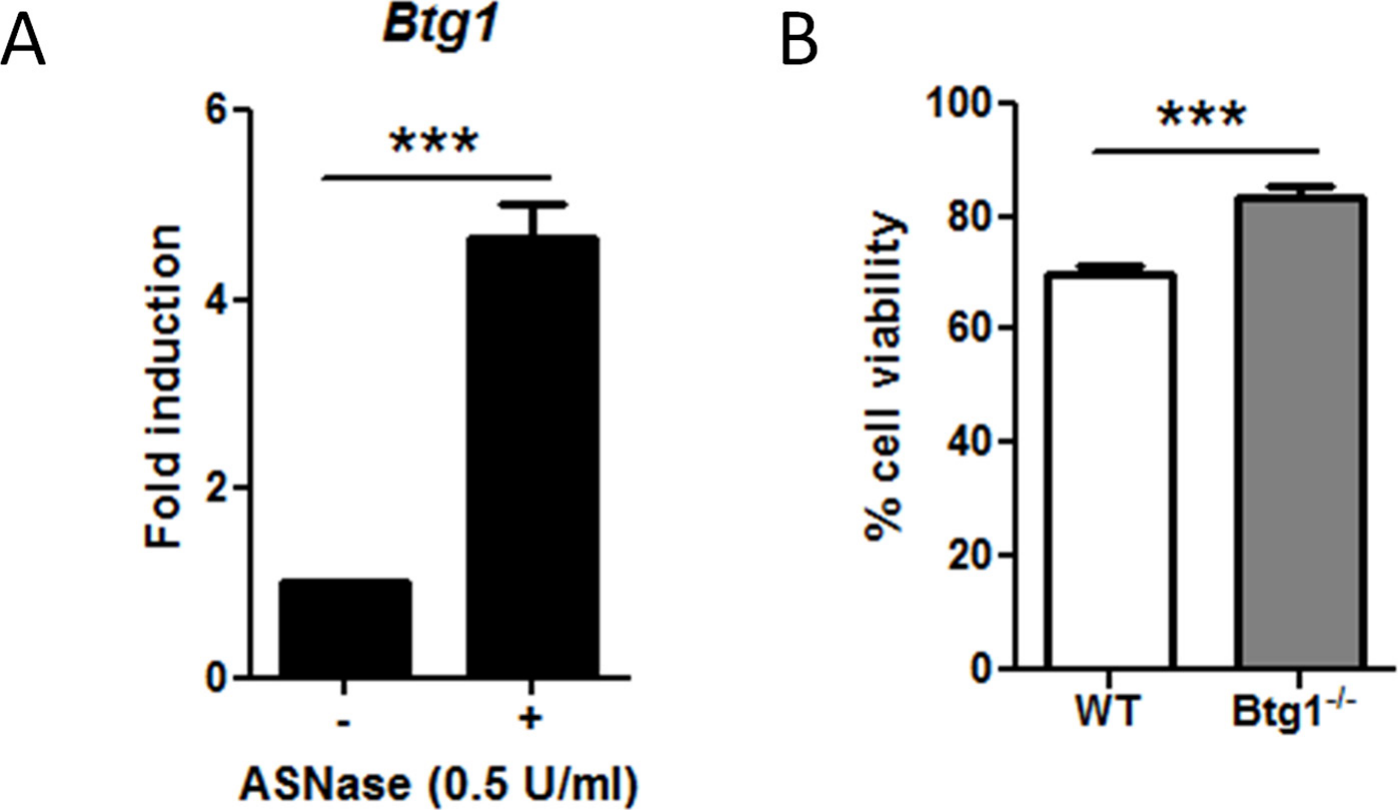
The absence of *Btg1* expression in mouse bone marrow-derived B-cell progenitors enhances cell survival in ASNase-treated cells (**A**) *Btg1* expression is upregulated upon ASNase treatment. WT CD19-positive bone marrow cells were treated with 0.5 U/ml ASNase for 48 h. *Btg1* mRNA levels were measured by qPCR and the relative expression compared to untreated cells (set to 1) is shown. Bars represent average data from four independent experiments ± SEM. (**B**) WT and *Btg1*^−/−^ CD19-positive bone marrow cells were treated with 0.5 U/ml ASNase for 48 h. The metabolic activity of the cells was measured by MTS assay and the relative cell survival compared to untreated cells (set as 100%) is shown. Bars represent average data from six independent experiments ± SEM. *p*-values are indicated with ***P < 0.001 (two-tailed *t*-test).

As our results demonstrate that BTG1 and ATF4 are important players in cellular stress adaptation in MEFs, we speculated that a similar mechanism takes place in other cell types. Since *BTG1* is frequently deleted in B cell progenitor-ALL, we investigated the involvement of BTG1 and ATF4 in cellular stress adaptation of CD19 positive mouse B-cell progenitors isolated from bone marrow. Due to unavailability of medium lacking of glutamine for growing bone marrow cells, we exposed the cells to Asparaginase (ASNase), a cornerstone drug in the treatment of ALL patients. ASNase acts by depleting asparagine from the blood, causing amino acid limitation in lymphocytes as well as leukemic blasts, which are uniquely sensitive to this drug as they lack the ability to synthesize asparagine. Similar to glutamine starvation, ASNase-induced asparagine depletion is known to activate ATF4 in an AAR-dependent manner [55]. Consistent with a role for BTG1 in modulating ATF4-mediated stress responses, we observed that *Btg1* mRNA was upregulated five-fold by ASNase treatment in these B-cell progenitors (Figure 5A). Moreover, we observed similar regulation of *BTG1* mRNA levels in human peripheral blood mononuclear cells (PBMCs) as well as leukemia cell lines treated with various stress-inducing agents (Supplementary Figure S4). Analogous to what we observed in the MEFs, B-cell progenitors derived from *Btg1* knockout mice showed a 20% increase in survival in comparison to WT cells in response to asparagine depletion (Figure 5B). We conclude from these experiments that, across different cell lineages, tumor suppressor BTG1 acts as an enhancer of ATF4 mediated stress responses, while a loss of BTG1 function promotes cellular survival in response to nutrient stress.

## DISCUSSION

*BTG1* is one of the genes recurrently affected by deletion or mutation in ALL or B-cell lymphoma cases, respectively [24, 25, 28, 29]. Furthermore, *BTG1* mRNA is downregulated in several solid tumors, including thyroid, lung, nasopharyngeal and breast cancer, while decreased expression is correlated with poor overall survival or increased cellular invasion [30–34]. While there is ample data to support the notion that BTG1 is a negative regulator of proliferation in tumor cells [19, 40–42], it remains unclear how *BTG1* deficiency contributes to tumorigenesis. Using primary cells (MEFs, progenitor B cells) derived from *Btg1*^−/−^ mice, we demonstrate that loss of *Btg1* is sufficient to promote adaptation and survival under drug-induced ER stress, nutrient starvation and amino acid limitation. Although BTG2 is highly similar to BTG1, both in structure and function [18], BTG2 expression or regulation does not appear to affect the response to these cellular stresses in our models. By analyzing gene expression in response to glutamine starvation, we identified ATF4 as the main transcription factor negatively affected by loss of BTG1 function. Glutamine is a major source of energy and an important metabolite for proliferating cells (e.g., fibroblasts, lymphocytes and enterocytes), and in addition to glucose metabolism, glutamine consumption is frequently elevated in rapidly growing tumor cells [46–48, 56]. Glutamine limitation increases ATF4 protein translation via the GCN2-eIF2α pathway [14, 15, 57]. Intriguingly, we observed that in our experimental model, only a subset of ATF4 target genes was dependent on BTG1 function. It is known that different subsets of ATF4 targets are activated in response to different types of stress stimuli [3]. Together, our results suggest a unique role for BTG1 in regulating the specificity of ATF4 target gene activation during nutrient deprivation.

More specifically, our results indicate that BTG1 dictates ATF4 function and specificity under nutrient limiting conditions, by controlling recruitment of ATF4 to the promoter of its target genes. This regulation is most likely achieved through BTG1 interacting with the arginine methyl transferase PRMT1. The BTG1-PRMT1 complex has previously been shown to mediate arginine methylation-mediated signaling events as well as differentiation [23, 36]. We demonstrate that PRMT1 binds to ATF4 in a BTG1-dependent manner and that PRMT1 methylates ATF4 *in vitro*. Although arginine 239 is the major target for PRMT1 activity, there may be additional arginine methylation sites in ATF4, since mutation in this residue does not completely abolish methylation of ATF4. Experiments using *Atf4*-deficient MEFs, complemented with either recombinant *ATF4-WT* or the hypo-methylated *ATF4-R239K*, revealed subgroups of ATF4 targets that are either critically dependent on ATF4 or require a threshold level of this transcription factor. In this system, we observed such a dependence for three ATF4 target genes *(Trb3, Fgf21, Slc6a9)*, and for each of these three genes, induction by ATF4 was less prominent in cells expressing ATF4-R239K. Similarly, pharmacological inhibition of PRMT1 negatively affects protein expression of ATF4 targets, underscoring an important role for PRMT1 in the regulation of stress-mediated gene expression.

We also established that *Btg1* deficiency promotes cellular stress adaptation to the protein drug ASNase in mouse bone marrow-derived B cell progenitors. Importantly, availability of asparagine is sufficient to overcome apoptosis induced by glutamine deficiency in tumor cells, implying that intracellular asparagine levels are critical determinants of the adaptive stress response in other malignancies as well [60]. As leukemic blasts frequently experience nutrient limitation (as in the case of ASNase administration), our findings suggests that cells lacking BTG1 may dampen ATF4-mediated stress responses, preventing the induction of apoptosis. Hence, we hypothesize that loss of BTG1 function, as it occurs during leukemic transformation, may provide leukemic blasts with a survival advantage, particularly when challenged with chemotherapy. Although the presence of *BTG1* deletions at diagnosis in most patient subgroups does not associate with unfavorable prognosis in pediatric B-ALL patients [61], the fact that clones carrying *BTG1* microdeletions are frequently preserved and arise *de novo* at relapse implies that such clones may show increased resistance to chemotherapy [26, 27]. There is growing evidence that cellular stress response pathways are exploited by tumor cells to sustain growth under unfavorable conditions or to resist therapy-induced apoptosis [62, 63]. Moreover, it was reported recently that the UPR arm of the ATF4 signaling cascade plays a key role in maintaining the cellular integrity of hematopoietic stem cells exposed to ER stress, by enabling these cells to either resolve stress or initiate apoptosis [13].

**Figure 6:**
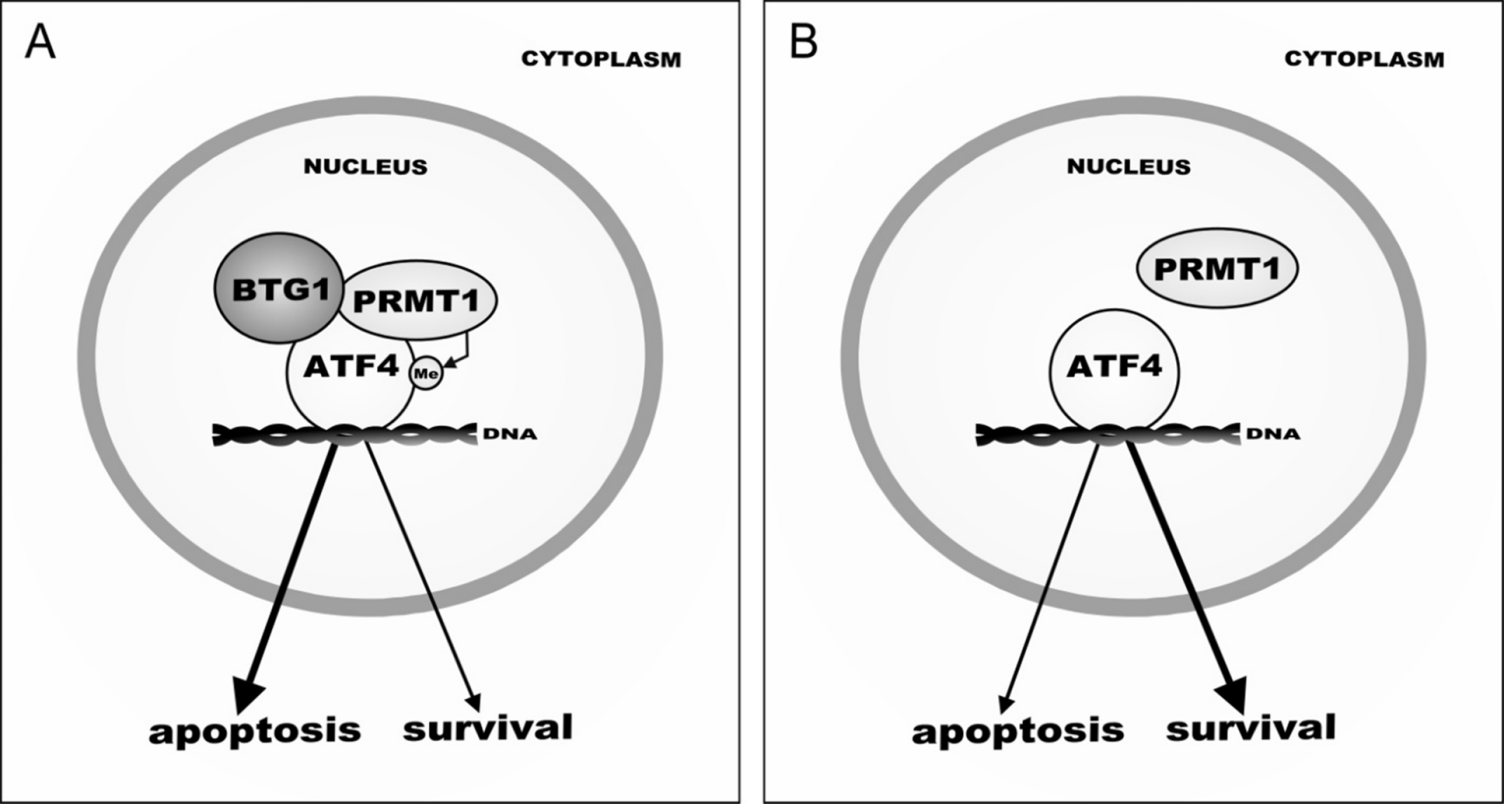
Model of BTG1-mediated ATF4 regulation during cellular stress (**A**) In response to sustained stress conditions, BTG1 recruits PRMT1 to methylate ATF4, - indicated by ‘Me’- which promotes transcription of a subset of ATF4 target genes, leading to increased apoptosis. (**B**) In the absence of BTG1, PRMT1 no longer binds to and methylates ATF4, shifting the balance from pro-apoptotic to pro-survival. As a consequence, loss of *BTG1* function promotes (tumor) cell survival.

Although several studies reported upregulation of ATF4 to be advantageous for tumor cells under unfavorable conditions [14, 38, 64], ATF4-mediated survival appears to be determined by both the level of ATF4 expression as well as the specificity of target genes activated by ATF4 [2, 15]. The fact that we see increased survival in *Btg1* knockout cells under cellular stress conditions, whilst ATF4 activity is dampened, should be seen in this context. The subset of ATF4 targets affected by *Btg1* loss is either implicated in the induction of apoptosis or stress-induced growth arrest. For instance, DDIT3 (C/EBP homology protein (CHOP)) and ATF3 are markedly upregulated in response to ATF4 activation, particularly under prolonged stress conditions, which eventually leads to cell death in normal as well as cancer cells [2, 39, 65, 66]. Similarly, TRB3 was shown to be responsible for neuroblastoma cell death upon glutamine starvation [10, 15, 65]. PPP1R15A is the mouse homolog of GADD34 (Growth Arrest and DNA Damage-Inducible 34), which is induced by ER stress, nutrient deprivation as well as other stress signals, leading to the induction of apoptosis [65, 67]. NDRG1 plays a role in stress-induced growth arrest and is required for p53-mediated apoptosis [68]. Therefore, we postulate that reduced activation of these ATF4 target genes, as observed in the *Btg1* knockout cells, improves adaptation to cellular stress by avoiding cell death. Overall, our findings identify BTG1 and PRMT1 as novel determinants of ATF4-mediated cellular stress responses (Figure 6). However, it remains to be investigated to what extent stress-induced apoptosis or salvage mechanisms are affected in tumors that show loss of BTG1 function.

## MATERIALS AND METHODS

### Cell culture

Generation of C57BL/6J *Btg1*^−/−^ and *Btg2*^−/−^ mice has been previously reported [20, 69]. Primary mouse embryonic fibroblasts (MEFs) were isolated by trypsinization of embryos dissected from day 13.5 of gestation, and their identities were verified by genotyping and quantitative real-time PCR (qPCR). SV40-immortalized WT and *Atf4*^−/−^ MEFs were described previously [70]. MEFs were cultured in DMEM containing Glutamax (Invitrogen) supplemented with 10% heat-inactivated fetal bovine serum (FBS; Greiner Bio-One), 1% penicillin/streptomycin (P/S; Invitrogen) and 55 μM b-mercaptoethanol (Gibco). The medium of immortalized MEFs was supplemented with 1% non-essential amino acid (Invitrogen). When indicated, glutamine-free DMEM (Invitrogen) supplemented with 10% dialyzed serum (Invitrogen), P/S and b-mercaptoethanol was used. Bone marrow (BM) cells were isolated from 8–12 week WT and *Btg1*^−/−^ mice using standard procedures, MACS-sorted using CD19 microbeads (Miltenyi Biotec) and co-cultured with OP9 stroma in Modified IMEM (Invitrogen) supplemented with 2% FBS, 1% P/S, b-mercaptoethanol, 0.3% w/v Primatone (Sigma) and 20 ng/ml IL7 (R&D Systems). The human B-ALL cell line (Nalm6) and the HEK293 cell line were purchased from ATCC and maintained in RPMI (Invitrogen) and DMEM, respectively, supplemented with 10% FBS and 1% P/S. Human peripheral blood mononuclear cells (PBMCs) were isolated from healthy donors using Ficoll-Paque PLUS (GE Healthcare Life Sciences) following manufacturer's protocol and maintained in RPMI supplemented with 10% FBS and 1% P/S.

### Plasmid construction and transient transfections

pCDNA3-ATF4 was generated by digesting pcDNA5/FRT/TO-ATF4-ORF [71] with *Eco*RI and the resulting ATF4 ORF was cloned into pCDNA3.1 using the EcoRI sites. FLAG-ATF4 was amplified from pCDNA3. 1-ATF4 and cloned into *Xho*I sites of pCDNA3.1. HA-BTG1 was amplified by PCR from pLZRS-HA-BTG1 [36] and cloned into pCDNA3.1 using the *Eco*RI and *Xho*I sites. Primers used for plasmid construction are listed in Supplementary Table S1. For transient transfection of HEK293, 5 × 10^6^ cells were seeded in a 10 cm dish and transfected with pCDNA3.1-FLAG-ATF4 and pCDNA3.1-HA-BTG1 using Polyethylenimine (PEI) (Brunschwig Chemie BV). For transient transfection of primary MEFs, 2 × 10^6^ cells were seeded in a 10 cm dish and transfected with pCDNA3.1-HA-BTG1 or empty vector using Viromer Red (Lipocalyx) according to manufacturer's instructions. MG132 proteasome inhibitor (Peptides International) was added 24 hours after transfection at a final concentration of 5 μM.

### Stress induction, cell viability and cellular toxicity assay

Human leukemia cell line and MEFs were treated with the following conditions: 2 μM thapsigargin (Sigma) for 24 h, 2 μg/ml tunicamycin (Sigma) for 24 h, glucose starvation (glucose-free DMEM (Invitrogen)) for 48 h, glutamine starvation (glutamine-free DMEM) for 24 h, and 2 U/ml Asparaginase (Takeda Nederland BV) for 48 h. CD19-positive B-cell progenitors from the bone marrow were treated with 0.5 U/ml Asparaginase for 48 h. As much as 5 × 10^3^ MEFs and 2 × 10^5^ bone marrow cells were seeded into a 96 well plate and cell viability was measured by CellTiter 96^®^ AQueous One Solution Cell Proliferation Assay (MTS) reagent (Promega) following manufacturer's protocol. For measurement of cellular toxicity, 2 × 10^4^ MEFs were incubated with indicated compounds in a 96 well plate and assayed with LDH Cytotoxicity Assay following manufacturer's instruction (Thermo Scientific).

### mRNA microarray and bioinformatics analysis

Total RNA was extracted from WT and *Btg1*^−/−^ MEFs using RNeasy Minikit (Qiagen) according to manufacturer's protocol. The quality control, RNA labeling, hybridization and data extraction were performed at ServiceXS B.V. (Leiden, The Netherlands). RNA concentration was measured using the Nanodrop ND-1000 spectrophotometer (Nanodrop Technologies). The RNA quality and integrity were determined using Lab-on-Chip analysis on the Agilent 2100 Bioanalyzer (Agilent Technologies, Inc.). Biotinylated cRNA was prepared using the Illumina TotalPrep RNA Amplification Kit (Ambion, Inc.) according to the manufacturer's specifications with an input of 200 ng total RNA. Per sample, 750 ng of the obtained biotinylated cRNA samples was hybridized onto the Illumina MouseRef-8 v2 (Illumina, Inc.). Gene expression data were normalized by the Robust Microarray Analysis (RMA) method using ArrayStar^®^ software (DNA STAR). To correct for background signals, we ruled out the genes with normalized expression below 3. Subsequently, the expression value of each gene after glutamine starvation was correlated to the expression level of the corresponding untreated sample, resulting in a fold change (FC) for each gene. We further defined a cut-off of ≥ |3| for the FC. The genes/mRNAs meeting these criteria from both WT and *Btg1*^−/−^ MEFs were analyzed using the Ingenuity Pathway Analysis software package (IPA, Ingenuity). For this study, we conducted IPA ‘transcription regulator’ analyses of the differentially expressed genes. In Table 1, the top five transcription regulators - i.e., the regulators with the lowest *P* values for enrichment of their targets within the differentially expressed genes and with a clear predicted activation state (Z-score ≥ |2|) - were shown. From this list, we generated a list of unique targets, i.e., genes that are differentially expressed in WT versus *Btg1*^−/−^ MEFs following glutamine starvation (Tables 2 and 3).

### Quantitative real-time PCR (qPCR)

RNA was isolated as described above. Subsequently, 1 ug of RNA was used for cDNA synthesis using iScript cDNA synthesis kit (Biorad) according to manufacturer's guidelines. cDNA was analyzed by qPCR using SYBR^®^ green (Applied Biosystems) on a CFX96™ Real Time PCR system (Biorad). Expression levels were normalized using the housekeeping gene HPRT (mouse genes) or TBP (human genes). Primer sequences used in qPCR experiments are listed in Supplementary Table S2.

### Co-immunoprecipitation (Co-IP)

MEFs were cultured in DMEM without glutamine or in the presence of 2 μg/ml tunicamycin for 24 hours to induce ATF4 expression. Cells were collected by trypsinization and lysed in RIPA buffer (150 mM NaCl, 1% NP40, 5 mM EDTA, 50 mM Tris, 0,5% deoxycholate, 0,1% SDS) supplemented with complete protease inhibitor cocktail mix (Roche). For Co-IP, lysates were incubated with antibody at 4°C overnight in a turning wheel. Subsequently, 25 μl of protein G Sepharose beads (GE healthcare) was added to the lysates and incubated at 4°C for 1 hour in a turning wheel. Protein-protein interactions were determined by immunoblot analysis.

### Immunoblot analysis

Cells were lysed in RIPA buffer supplemented with complete protease inhibitor cocktail mix. Equal amount of proteins were separated by SDS-PAGE and subsequently transferred to 0.45 μm PVDF membrane (Thermo Scientific). Antibodies used were CHOP (D46F1), ATF-4 (D4B8), PRMT1 (F339), PARP (#9542) from Cell Signaling Technology; CREB-2/ATF4 (C-20), ATF-3 (C-19), GAPDH (H-12), β-actin (AC-15) from Santa Cruz Biotechnology; HA tag (3F10) from Roche; FLAG tag (F3165) from Sigma; anti-rabbit and anti-mouse HRP from Dako; anti-rabbit IgG light chain HRP (ab99697) from Abcam. Protein expression was detected by ECL™ Western Blotting Prime Detection Reagent (GE Healthcare) and visualized using Fluorochem E (Westburg, Cell Biosciences) or autoradiography.

### Chromatin Immunoprecipitation (ChIP)

Immunoprecipitated DNA fragments for ChIP were prepared from primary WT and *Btg1*^−/−^ MEFs depleted for glutamine for 16 h, followed by crosslinking with 1% formaldehyde for 10 min and ChIP using anti-ATF4 (D4B8, Cell Signaling Technology) antibody. Immunoprecipitation without an antibody served as negative control. For each target gene, qPCR was performed using primers that recognize the ATF4 binding site (upstream) and a control region around 1.5 kb further (downstream). ChIP-qPCR primers are listed in Supplementary Table S3. Promoter occupancy was calculated by correcting the percentage recovery of each target gene with that of albumin.

### Generation of ATF4 methylation mutants and *in vitro* methylation assay

Deletions of the C-terminus region of ATF4 and nucleotide substitutions to convert arginine residues of ATF4 into lysine (Figure 3C) were introduced by overlap extension PCR using *Taq* polymerase (Invitrogen) following manufacturer's protocol. The primers used for overlap extension PCR are listed in Supplementary Table S1. WT and methylation mutants of ATF4 were cloned into *Eco*RI sites of pGEX1N (GE Healthcare Life Science). These GST-tagged proteins were produced in *E. coli* BL21 pLysS DE3 Rosetta2 (Millipore) and isolated using the B-PER GST spin purification kit (Pierce) according to manufacturer's instruction. As much as 1 μg of purified recombinant PRMT1 (ProSpec Bioscience) was incubated with 5 μg of purified GST-fusion ATF4 in the presence of radioactive methyl donor [3H-methyl] -S-Adenosyl Methionine (55–85Ci (2.03–3.15TBq)/mM; GE Healthcare Life Science) in a total of 30 μl of PBS for 2 h at 30°C. The reaction was stopped by addition of SDS-PAGE sample buffer and analyzed by SDS-PAGE, blotting and autoradiography.

### Retroviral transduction

Both WT and R239K ATF4 were cloned into the *Eco*RI sites of pBABE-puro [72]. Retroviruses containing WT and R239K ATF4 as well as empty vector (EV) were produced in Phoenix ecotropic cells using Lipofectamine 2000 (Invitrogen) according to manufacturer's guidelines. Immortalized *Atf4*^−/−^ MEFs were subsequently transduced with these three retroviruses and subjected to antibiotic selection with 1 ug/ml puromycin (Sigma).

### PRMT1 inhibition

PRMT1 methyl transferase activity was inhibited by adding AMI-1 (Santa Cruz Biotechnology, sc-205928) at a concentration of 50 uM for indicated times, in the absence of glutamine.

### Statistical analysis

Statistical data are expressed as mean ± SEM. Statistical differences were determined with 2-sided, paired or unpaired Student's *t* tests as indicated in the text; *p* values < 0.001 (***), < 0.01 (**) or < 0.05 (*) were considered statistically significant.

### Accession numbers

Illumina bead array expression data have been submitted to the NCBI Gene Expression Omnibus database (http://www.ncbi.nlm.nih.gov/geo/), accession code: GSE63642.

## SUPPLEMENTARY MATERIALS FIGURES AND TABLES


