# Supplementary material for: Tumor suppressor BTG1 promotes PRMT1-mediated ATF4 function in response to cellular stress
Source: Oncotarget. 2015 Dec 9;7(3):3128–43. doi: 10.18632/oncotarget.6519 (PMC4823095; doi:10.18632/oncotarget.6519)
Supplement: Supplementary file 1 [file oncotarget-07-3128-s001.pdf]

Tumor suppressor BTG1 promotes PRMT1-mediated ATF4 function in response to cellular stress

Supplementary Materials

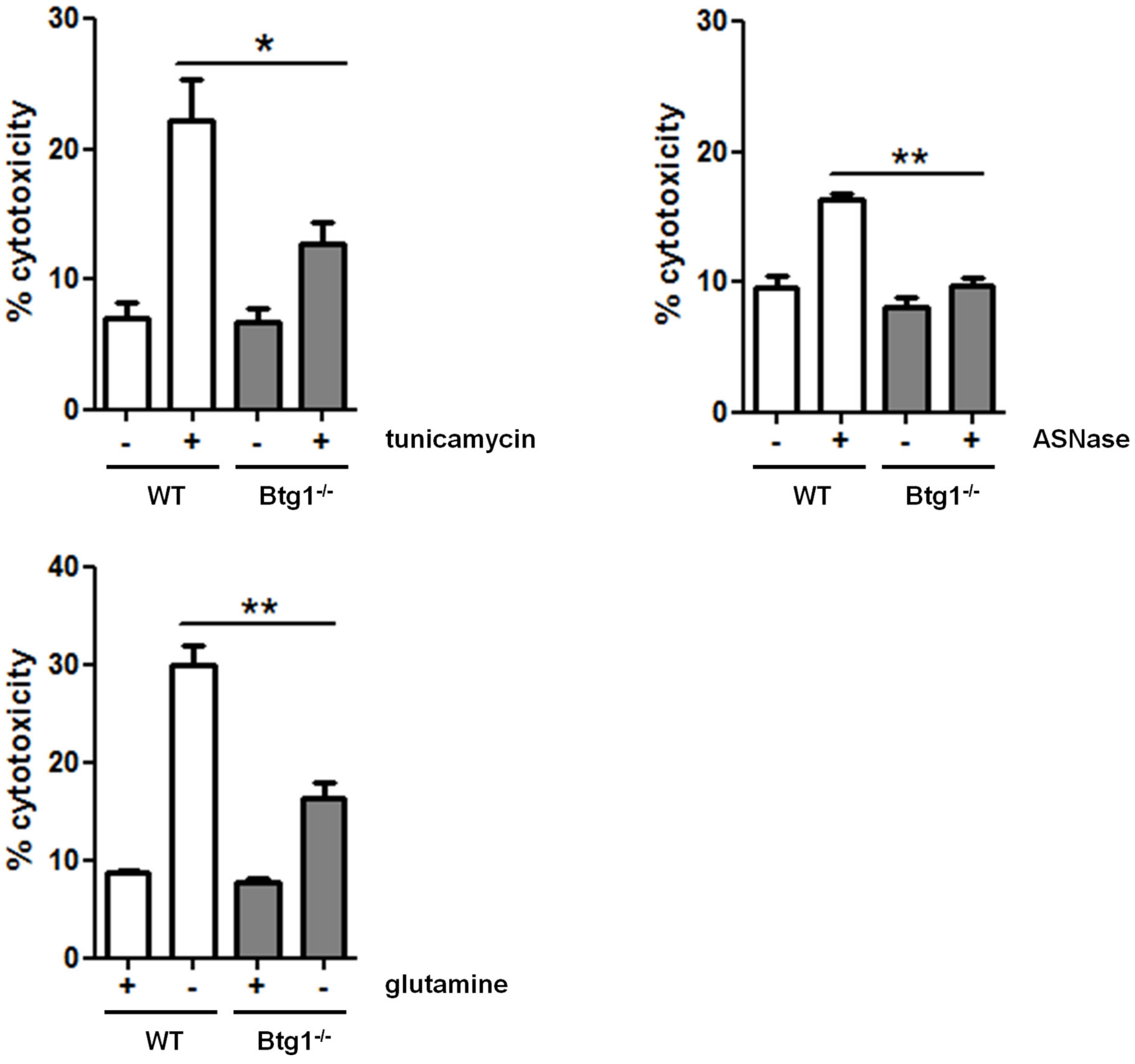

Supplementary Figure S1: Loss of BTG1 decreases cellular toxicity upon stress treatments. WT and *Btg1*$^{-/-}$ MEFs were treated with indicated stressors and the level of cellular toxicity, as indicated by lactate dehydrogenase (LDH) released into the media from damaged cells, was determined. Bars represent average data from three or four independent experiments ± SEM. *P*-values are indicated with $**P < 0.01$ and $*P < 0.05$ (two-tailed *t*-test).

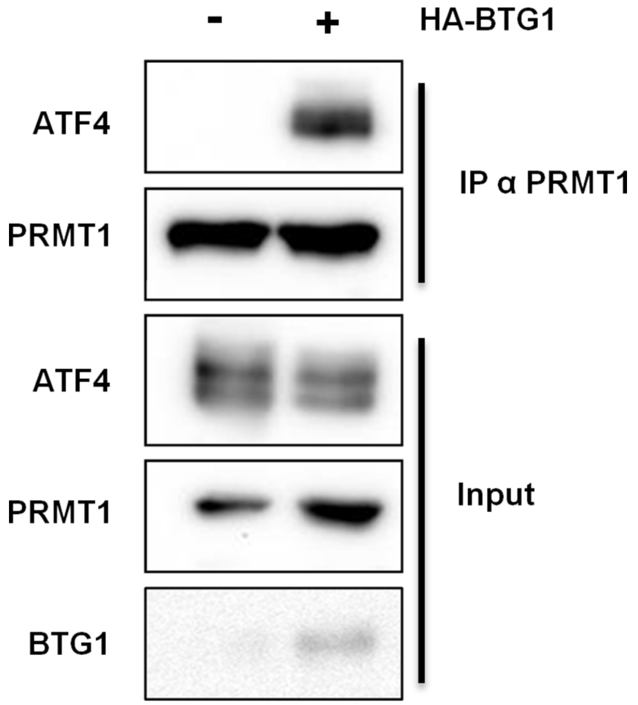

Supplementary Figure S2: Re-expression of BTG1 in *Btg1*$^{-/-}$ cells rescues PRMT1-ATF4 interaction. Primary *Btg1*$^{-/-}$ MEFs were transfected with an expression plasmid encoding HA-BTG1 or with an empty vector control. Cells were treated for 24 hrs with 2 μg/ml tunicamycin to induce expression of endogenous ATF4 and with 5 μM of the proteasome inhibitor MG132 to facilitate detection of the highly unstable BTG1 protein. Protein lysates were generated and subjected to immunoprecipitation (IP) using an anti-PRMT1 antibody. Immunoblot demonstrates expression HA-BTG1 using an anti HA antibody.

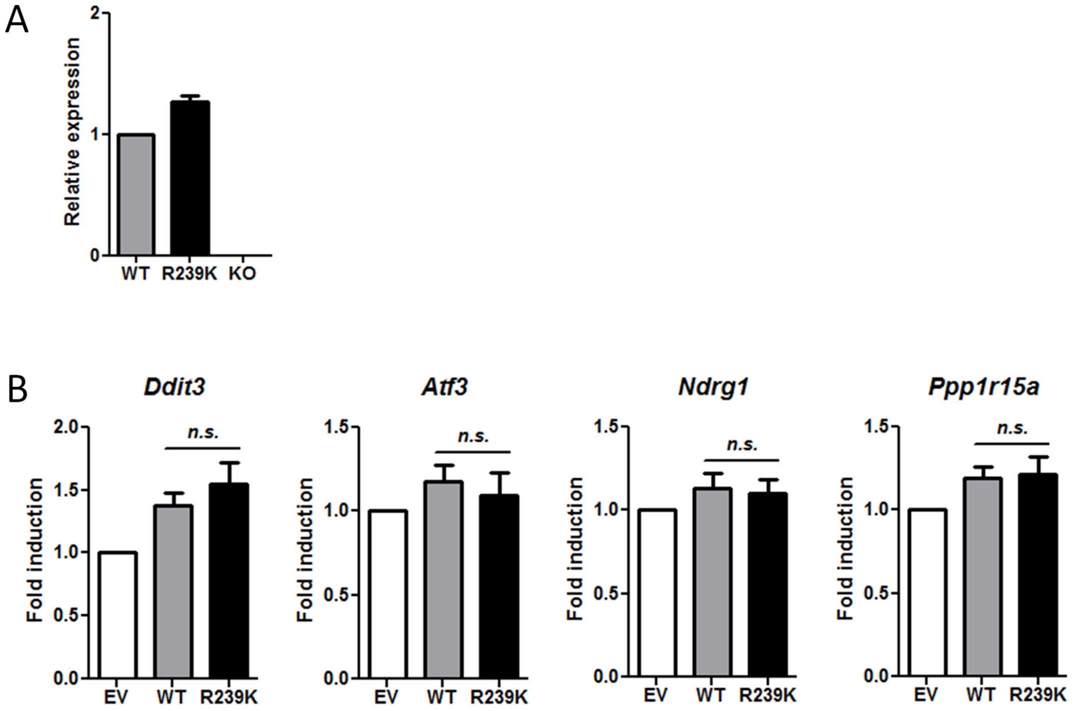

Supplementary Figure S3: Loss of PRMT1-mediated methylation at residue R239 does not affect the regulation of a subset of ATF4 targets. (**A**) Both *ATF4-WT* and *ATF4-R239K* are equally expressed at mRNA level. *ATF4-WT* and *ATF4-R239K* as well as an empty vector control (EV) were retrovirally transduced into immortalized *Atf4*$^{-/-}$ MEFs. Cells were subjected to qPCR to measure the ATF4 trancript levels (expression level of WT is set to 1). (**B**) EV, *ATF4-WT* and *ATF4-R239K* complemented MEFs were subjected to qPCR to measure expression of ATF4 target genes identified by gene expression analysis (Table 3). Data is presented as fold induction of mRNA (expression level of untreated EV MEFs were set to 1). Bars represent average data from four independent experiments ± SEM and *P*-values were calculated by two-tailed *t*-test.

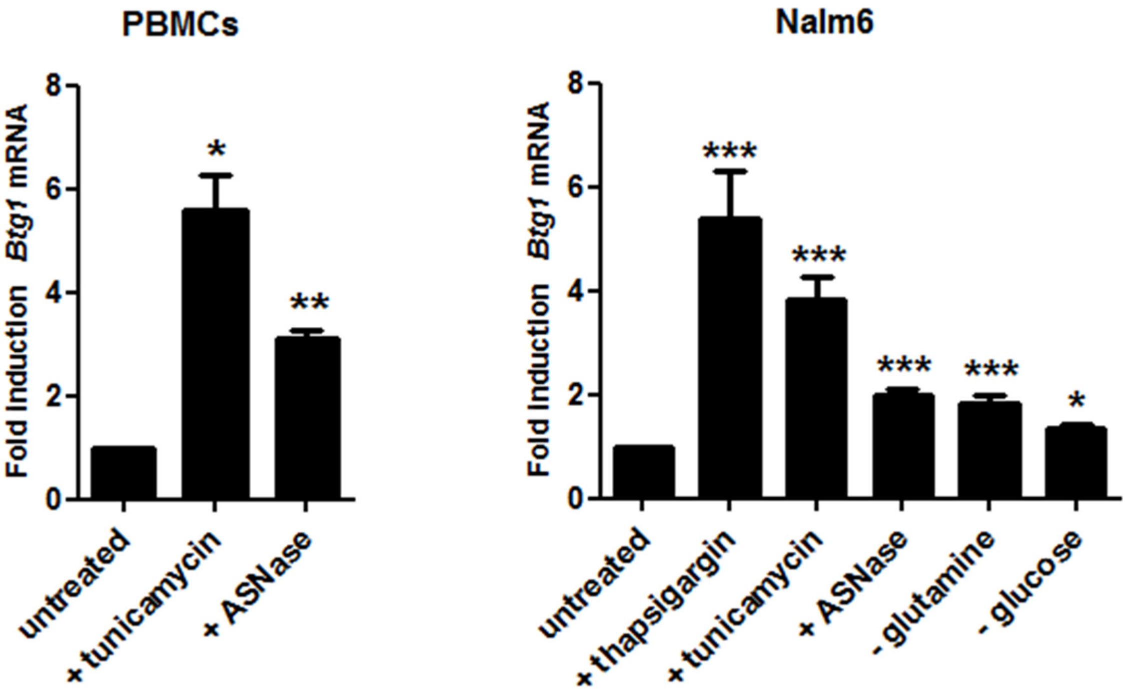

Supplementary Figure S4: *BTG1 mRNA* expression is upregulated in human hematopoietic cells. Human PBMCs and B-ALL cell lines (Nalm6) were treated with various stressors and *BTG1* mRNA levels relative to untreated cells are shown. Bars represent average data from two (PBMCs) and three (Nalm6) independent experiments ± SEM. *P*-values are indicated with ****P* < 0.001, ***P* < 0.01 and **P* < 0.05 (two-tailed *t*-test).

Supplementary Table S1: PCR primers used for plasmid constructions

Primer	5′→ 3′
FLAG-ATF4 fwd	GCCTCGAGACCATGGACTACAAAGACGATGACGATAAAACCGAAATGAGCTTCCTGAG
FLAG-ATF4 rev	CGCCTCGAGCTAGGGGACCCTTTTCTTCC
HA-BTG1 fwd	GCGAATTCGACCATGGCTTACCCATACGATGTTCCAGATTACGCTCATCCCTTCTACAC CCGGG
HA-BTG1 rev	GCCTCGAGTTAACCTGATACAGTCATCATATTG
GST-EcoRI-ATF4-fw	GCGGAATTCACCATGACCGAAATGAGCTTC
ATF4-XhoI-rev	CGCTCGAGTCACTAGGGGACCCTTTTCTTCCCCCT
ATF4 Δ321–351 rev	CGCTCGAGTCACTATAGAGCCTCGTTCTTCTT
ATF4 Δ278–351 rev	CGCTCGAGTCACTAGACCTCTTCTATCAAATC
R239 + 244K fwd	CCCCTCTACCAAGGGCTCTCCAAATAAGAGCCTCCC
R239 + 244K rev	GGGAGGCTCTTATTTGGAGAGCCCTTGGTAGAGGGG
ATF4 R239K fwd	CCTCTACCAAGGGCTCTCC
ATF4 R239K rev	GGAGAGCCCTTGGTAGAGG
ATF4 R244K fwd	CTCCAAATAAGAGCCTCC
ATF4 R244K rev	GGAGGCTCTTATTTGGAG
ATF4 R257K fwd	TCTGCCAAGCCCAAACCTTA
ATF4 R257K rev	TAAGGTTTGGGCTTGGCAGA

Supplementary Table S2: Primers used for qPCR

Primer	Forward (5′→ 3′)	Reverse (5′→ 3′)
mouse primers		
Ddit3	CAGAGCCAGAATAACAGCCG	CCAAGGTGAAAGGCAGGGAC
Atf3	GAGGATTTTGCTAACCTGACACC	TTGACGGTAACTGACTCCAGC
Trb3	CCACAGGCACAGAGTACACC	GATGTAAAGGAGCCGAGAGC
Slc6a9	AAAAGGTGCCAAAGGGATGTT	GTCAGTACAAACTCGATCTGGTT
Ndrg1	ATGTCCCGAGAGCTACATGAC	CCTGCTCCTGAACATCGAACT
Fgf21	CTGCTGGGGGTCTACCAAG	CTGCGCCTACCACTGTTCC
Ppp1r15a	GAGGGAC GCCCACAACTTC	TTACCAGAGACAGGGGTAGGT
Atf4	CCTGAACAGCGAAGTGTTGG	TGGAGAACCCATGAGGTTTCAA
Btg1	CCACCATGATAGGCGAGATCG	CTGGGAACCAGTGATGTTTG
Btg2	GGGTTTCCTCTCCAGTCTCC	GATACGGATACAGCGATAGC
Hprt	GGGGGCTATAAGTTCTTTGCTGACC	TCCAACACTTCGAGAGGTCCTTTTCA
human primers		
DDIT3	CCACCATGATAGGCGAGATCG	CTGGGAACCAGTGATGTTTG
ATF3	GGGTTTCCTCTCCAGTCTCC	GATACGGATACAGCGATAGC
TRB3	TGTCTTCGCTGACCGTGAGAGG	ACGCGTGCTTGTCCCACAGGGA
SLC6A9	CAGATCGAGTTTGTACTGACGAG	GCGATAGCAGAGGTATGGGAAG
NDRG1	CTCCTGCAAGAGTTTGATGTCC	TCATGCCGATGTCATGGTAGG
FGF21	GCCTTGAAGCCGGGAGTTATT	GTGGAGCGATCCATACAGGG
PPP1R15A	ATGATGGCATGTATGGTGAGC	AACCTTGCAGTGTCCTTATCAG
ATF4	CCTCCGAATGGCTGGCTGTGGA	CAGGGCATCCAAGTCGAACTCC
BTG1	AGCGGATTGGACTGAGCAG	GGTGCTGTTTTGAGTGCTACC
TBP	GCACAGGAGCCAAGAGTGAA	ACATCACAGCTCCCCACCAT

Supplementary Table S3: qPCR primers for ChIP assay

Primer	Forward (5′→ 3′)	Reverse (5′→ 3′)
upstream primers		
Ddit3	GGGCAGACAAGTTCAGGAAG	ATGATGCAATGTTTGGCAAC
Atf3	GGTCTCCACCCACCTTTTG	CTCGCTGAGTGAGACTGTGG
Fgf21	ACTAAGGTGAAGATCCCAACCTCC	TCCCACTCCTGACGCGTGATATTT
Albumin	TGCCATTGGGTTAGAGAAATG	TGGAAGAAGACCTTGTCCTGA
downstream primers		
Ddit3	AAAAACAAAAGCAGGGCAGA	TCCTAAAGATACCGGCATGG
Atf3	GACCAGGTTCCCAGAGTGAA	TACCTGGCACCCCTCATAGA
Fgf21	TTCTGGGAACCTCACAGCTCAACT	GCTGCCTTGGAATCACCCAAACTT
